# The folds and faults kinematic association in Zagros

**DOI:** 10.1038/s41598-022-12337-8

**Published:** 2022-05-19

**Authors:** Mohammad Ali Ghanbarian, Reza Derakhshani

**Affiliations:** 1 Department of Earth Science, College of Science, Shiraz University, Shiraz, Iran; 2 Department of Geology, Shahid Bahonar University of Kerman, Kerman, Iran; 3 Department of Earth Sciences, Utrecht University, Utrecht, The Netherlands

**Keywords:** Tectonics, Structural geology

## Abstract

The Zagros orogenic belt, one of the most prominent and famous collisional belts in the central part of the Alpine-Himalayan orogenic chain, is located between the southern margin of the Central Iranian microcontinent and the northern margin of the Arabian plate. The structural architecture and folds and faults relationships of a significant segment of the south-central part of the Zagros’ hinterland are investigated in this study through stereoscopy of aerial photographs, interpretations of satellite images, consideration of the major ground topographic variations, and field research. This research found that there must have been at least two major deformation events: (1) a ductile phase, which is older than the Eocene, and (2) a semi-brittle deformation stage, which is younger than the early Miocene and is represented by thrusting, folding, and strike-slip faulting. The presence of numerous fault-related folds and fold-accommodation faults in this area demonstrates the close kinematic relationship between folding and faulting. Based on the topographic changes, a major hidden tear fault and a basement hidden back thrust, which play important roles in the architecture of the area, have been suggested.

## Introduction

Explaining the structural architecture and considering the faults and folds relations in an area in the Zagros Hinterland Fold-Thrust Belt (ZHFTB) is the purpose of this paper. Folds and faults, as representatives of separate ductile and brittle deformations, were previously thought to only form in completely different tectonic settings^1–4^. However, these common structures can also develop simultaneously in a geodynamic environment as a result of kinematically related tectonic processes^5–8^. The fold-accommodation faults^7,9,10^ and the fault-related folds^5,11–13^ are the two broad categories of structures that illustrate the complete kinematic relationship between faults and folds. The fault-related folds in the study area have been explained by Sarkarinejad and Ghanbarian^14^. The present study aims to investigate the fold-accommodation faults, present the outlines of the structural architecture of the study area which is a part of the Zagros orogenic core, and add some further information about the fault-related folds of the area.

### Geological setting

**Figure 1:**
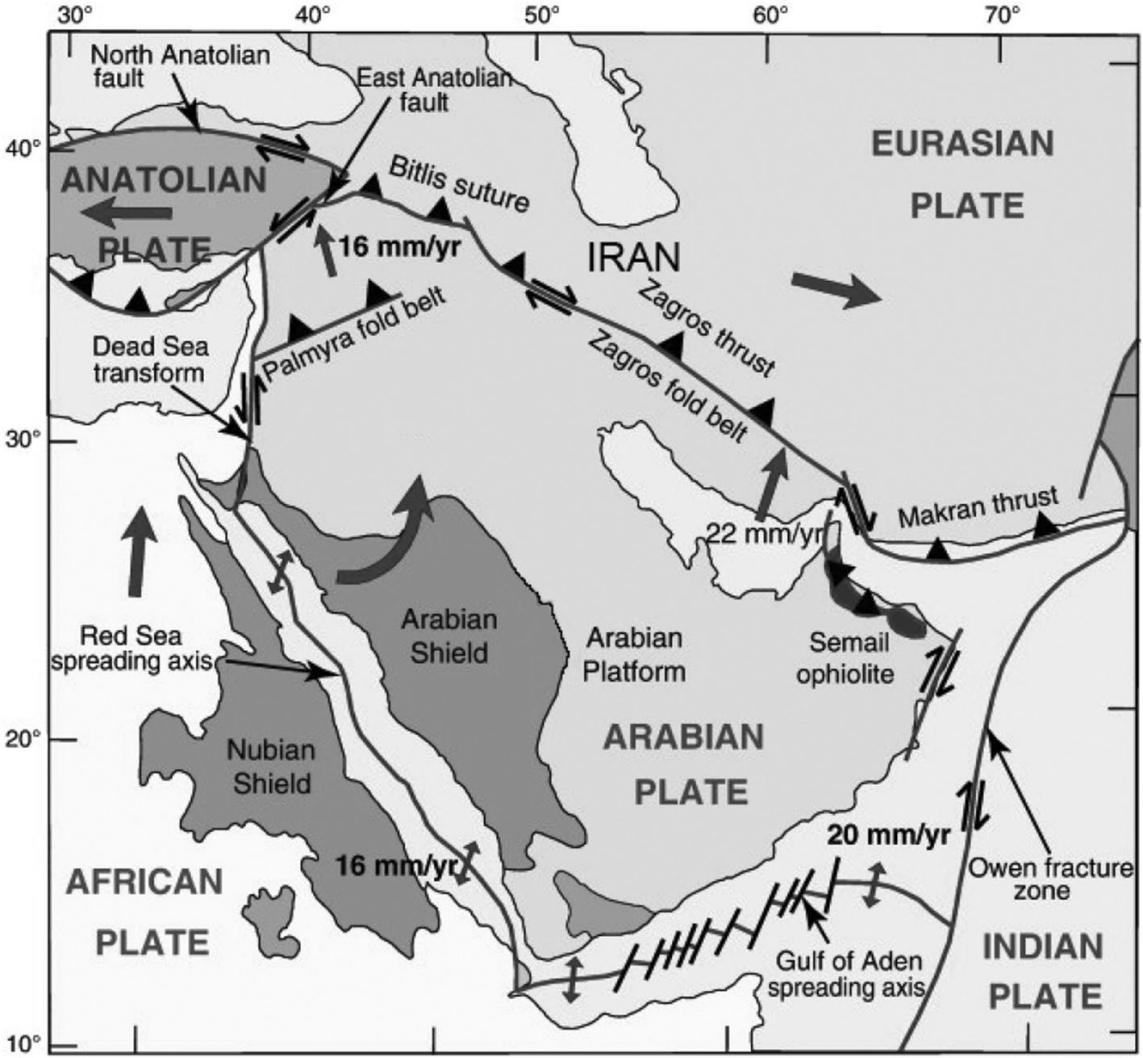
A simplified map showing the situation of the Zagros and the Eurasian and Arabian plates. In this schematic figure, plate boundaries, approximate plate convergence vectors, and some key geologic features are mapped for a better understanding of the tectonic situation of the region^31^.

The Zagros, as one of Iran's most major geological zones, has always been studied from a variety of viewpoints^15–18^. The Zagros orogenic belt consists of SW-verging mountains formed as a result of the long-standing and continuous subduction of the Neo-Tethyan oceanic lithosphere from the Jurassic to the Cenozoic and the subsequent collision of the Arabian plate with the Central Iranian microplate in Cenozoic^19–24^ (Fig. 1). This collisional belt, which has been the subject of several studies on its landscape evolution and deformation at regional and local scales, is approximately 2000 km long and comprises some parallel subdivisions^25–30^.

### Exposed stratigraphic units of the study area and its surroundings

**Figure 2:**
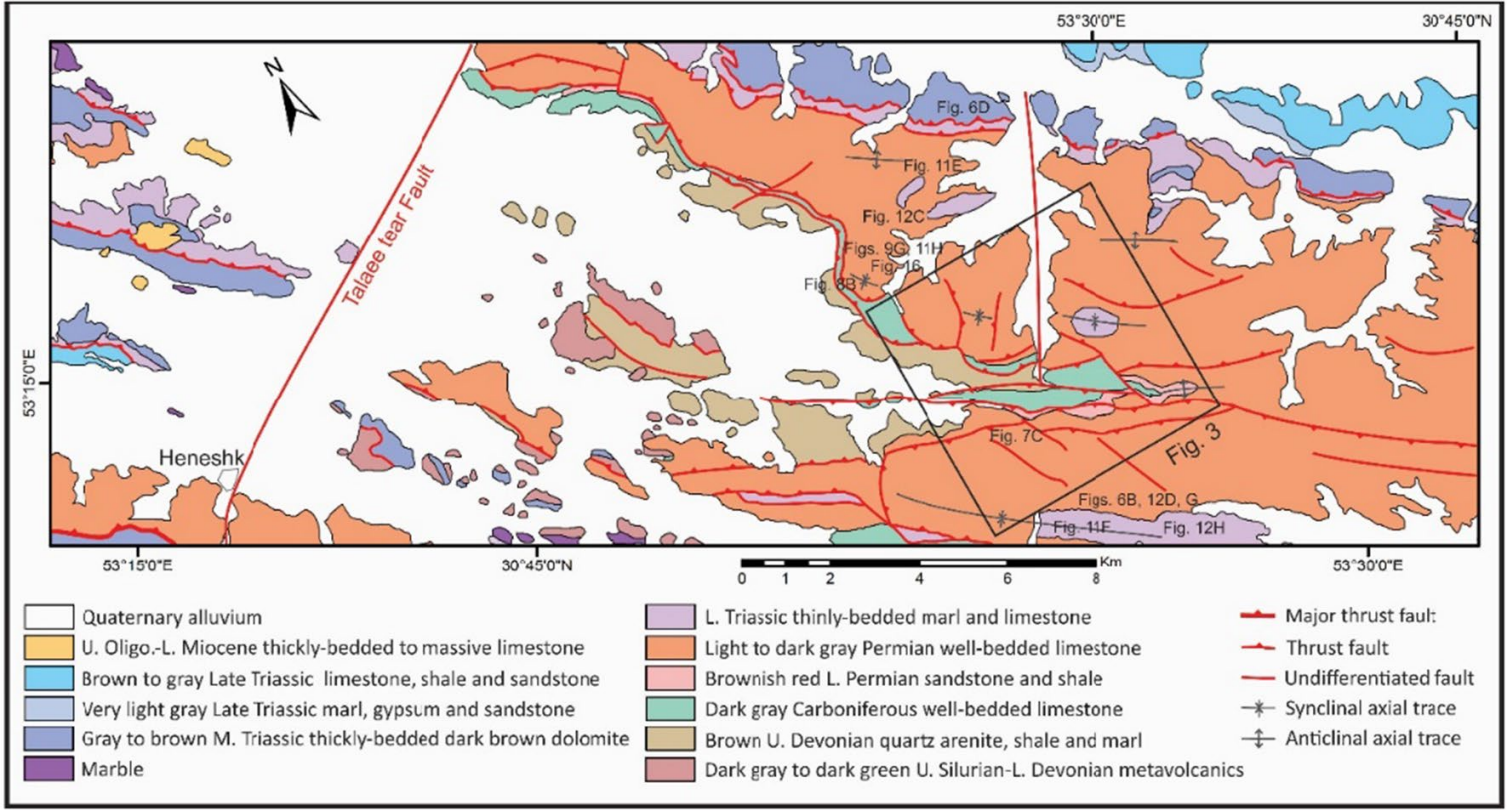
The geological map of the study area prepared after field working by authors using CorelDraw 2018 and ArcGIS 10.5.

**Figure 3:**
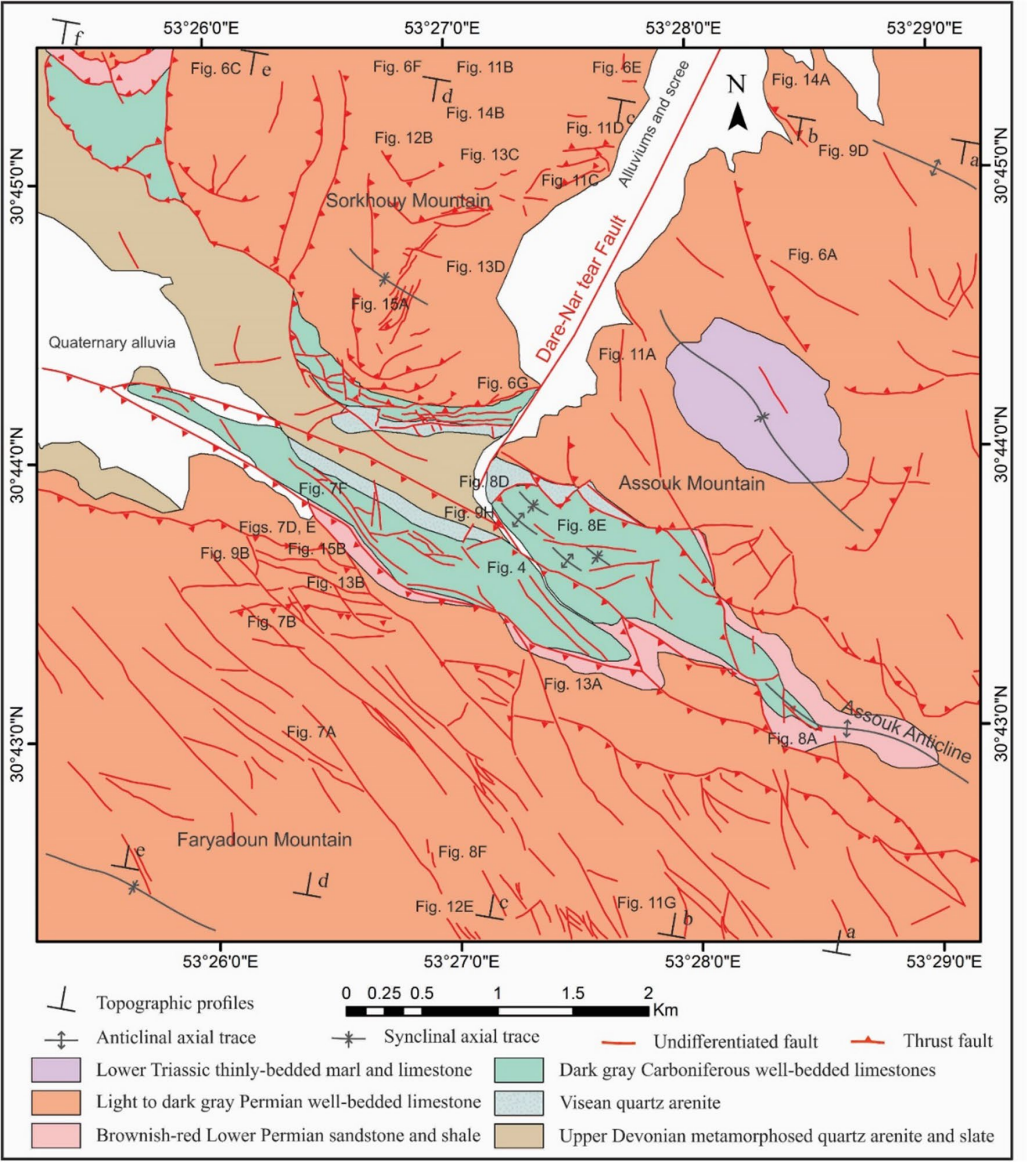
The geological map of the Faryadoun and Dare-Nar area prepared after field working by authors using CorelDraw 2018 and ArcGIS 10.5.

**Figure 4:**
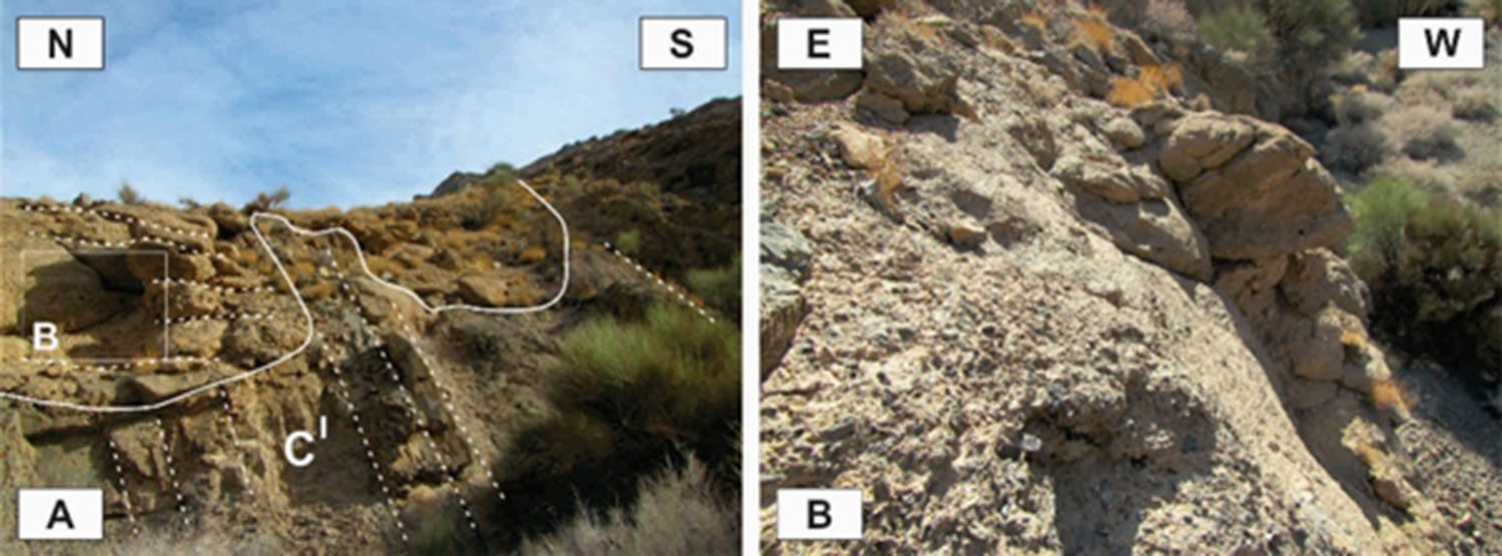
A horizontal conglomerate unit on the sub-vertical layers of the Carboniferous well-bedded limestones (C^l^) in the southernmost part of Dareh-Nar valley. (Photos are taken by Mohammad Ali Ghanbarian).

All of the exposed stratigraphic units of the study area (Figs. 2, 3) and some of the important units of its surroundings are explained below. The Upper Devonian gray metaterrigenous unit (metamorphosed quartz arenite and slate) (D^sh^) is one of the oldest exposed stratigraphic units in the study area, outcropped between the Sorkhouy Mountain and the Faryadoun Mountain (Fig. 3). This unit is overlain by the Visean quartz arenite unit (C^Q^), which is at the base of the dark gray Carboniferous well-bedded limestones (C^l^). Light to dark gray Permian well-bedded limestone (P^l^) is the next unit, with the brownish-red Lower Permian sandstone and shale (P^s^) at the base. Permian limestone (P^l^) forms the most widespread outcrops in the region. The Lower Triassic thinly-bedded marl and limestone (Tr^l^) are the younger exposed units in this area (in the Assouk Mountain). All of the mentioned units in the study area are influenced by low-grade metamorphism. The Upper Oligocene-Lower Miocene thickly-bedded to massive limestone overlies the Triassic units in the Pouzesiah Mountain (northwest of the study area) with angular unconformity^14^. There is a horizontal conglomerate unit on the sub-vertical layers of the Carboniferous limestones (C^l^) in the southernmost part of the Dareh-Nar valley (Fig. 4A). This unit has not been reported in the previous maps^32–34^ and articles^14,30^ of the study area. The particles of this unit are not well sorted (Fig. 4B), but the rock is very well cemented and compact. The granules, pebbles, cobbles, and boulders of this unit are angular and mostly composed of the limestones of the Carboniferous and Permian periods, as well as sandstone, and quartz arenites of the Visean.

### Data and method

In order to investigate the structural architecture and particularly the faults and folds relations in the study area, the vertical aerial photographs at 1:50,000 scale, taken from the archives of the Department of Earth Sciences at Shiraz University, and the Landsat 8 satellite (Google Earth) images were considered. Utilizing this data, map-scale structures (e.g., macroscopic faults and folds) have been carefully detected and mapped (Figs. 2, 3). While large-scale structures form the structural framework of the ZHFTB, mesoscopic ones are very valuable because, since the early twentieth century, it has been accepted as Pumpelly’s rule that the characteristics of map-scale architecture can be cleared up by surveying more plentiful and accessible associated minor structures^35^. Therefore, intensive field campaigns were conducted in the various parts of the study area, and outcrop-scale structures, including mesoscopic folds, different kinds of mesoscopic faults, and shear zones, were studied. Aerial photography becomes significantly more effective when a stereoscope is used in the field to gain a better understanding of field relationships. Using this advantage, it became clear that the mean elevation of the Faryadoun Mountain and its northern area (Dareh-Nar valley and its adjacent area) has changed significantly. As a result, approximately six kilometers of NNE-SSW topographic profiles have been constructed in this area to study this topographic contrast.

## Results

### Structural characteristics of the study area

**Figure 5:**
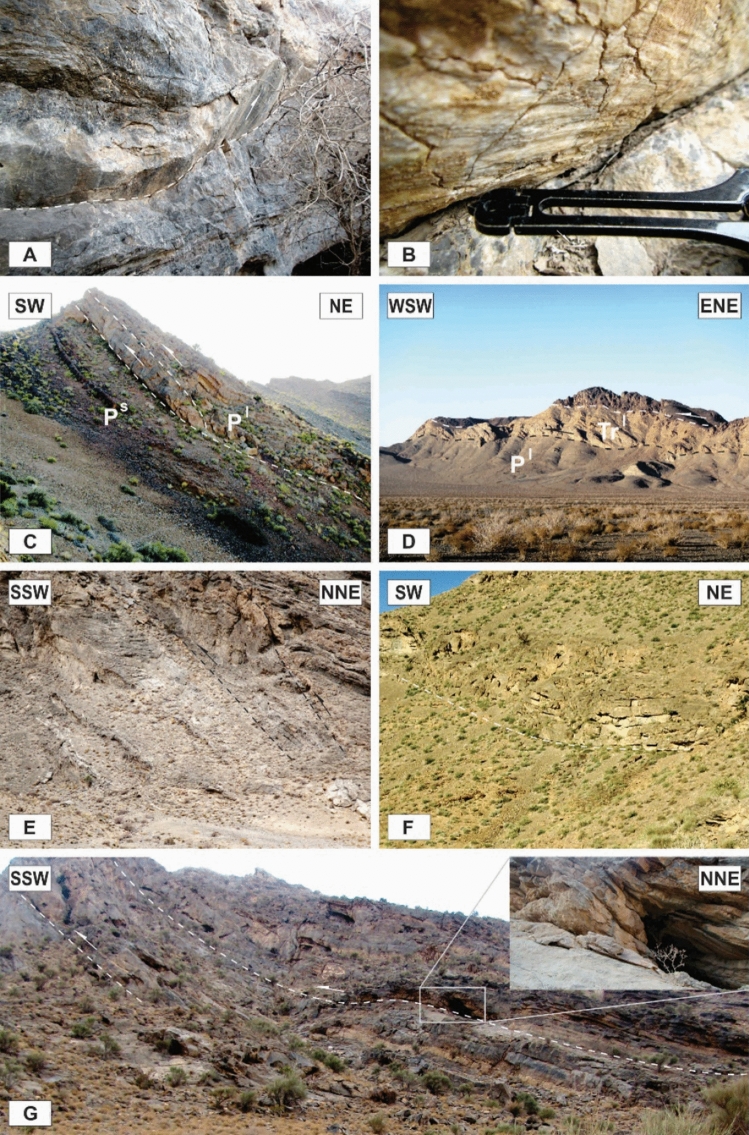
Some field photographs of two examples of faults’ planes and slickenlines (**A**,and **B**), and the map-scale thrusts and reverse faults (**C**–**G**) of the study area. The compass needle is 55 mm long and toward N. (Photos are taken by Mohammad Ali Ghanbarian).

**Figure 6:**
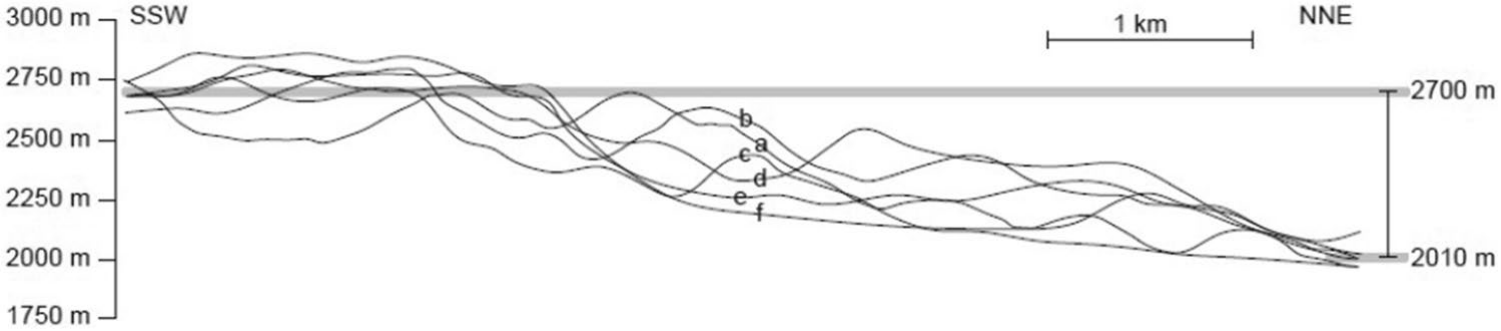
Six about 6 km long NNE–SSW topographic profiles have been constructed in the study area which shows a significant change (about 700 m) in the mean elevation between the Faryadoun Mountain and its northern area. See Fig. 3 for the locations.

**Figure 7:**
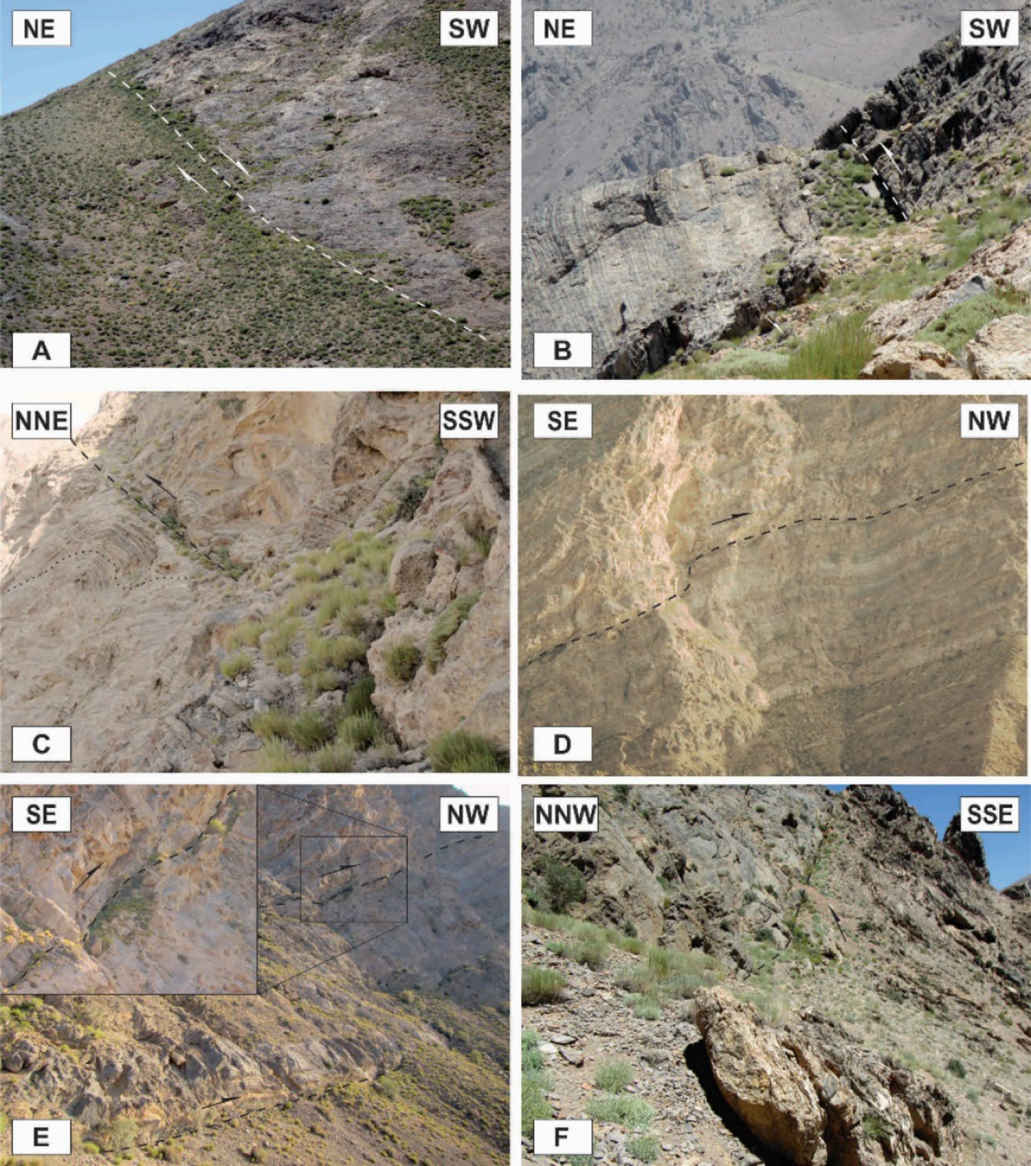
Annotated field photograph of a map-scale strike-slip fault (**A**) and several map-scale back thrusts of the study area (**B**–**F**). (Photos are taken by Mohammad Ali Ghanbarian).

The tectonic structures in the area occurred on a wide range of scales. The characteristics of map-scale structural architecture are formed by the large-scale structures that have been presented on the detailed maps of the study area (Figs. 3, 4). The structures that play the most important roles in the tectonic framework of the study area are the various kinds of faults, folds, and shear zones. In this study, the faults were recognized from topographic variations, strike separations, and the bedding attitudes or lithological characteristics observed during the field investigations, as well as from aerial photographs stereoscopy, satellite (Google Earth) image interpretations, and topographic profile considerations. Vertical aerial photographs have been used as a basis for field mapping, plotting dips, strikes, attitudes of faults, and contacts. The characteristics of the map-scale structures can be cleared up by surveying more reachable abundant outcrop-scale structures. In some cases, faults’ planes and slickenlines were observed during field campaigns (Fig. 5A,B). The surface exposures of the faults are more frequently observed in the more competent units (e.g., Carboniferous and Permian limestones) than in the more erodible ones (e.g., Upper Devonian metaterrigenous unit and Lower Triassic marls and limestones). The faults are one of the most abundant structures in the Dareh-Nar and Faryadoun areas (Figs. 5, 7). The various kinds of faults developed in the different parts of the study area. Due to their abundance, most of the contacts between lithological units are fault contacts. There is a big difference in the scale of the faults in the range of map-scale to outcrop-scale (Fig. 6).

### Map-scale faults

**Figure 8:**
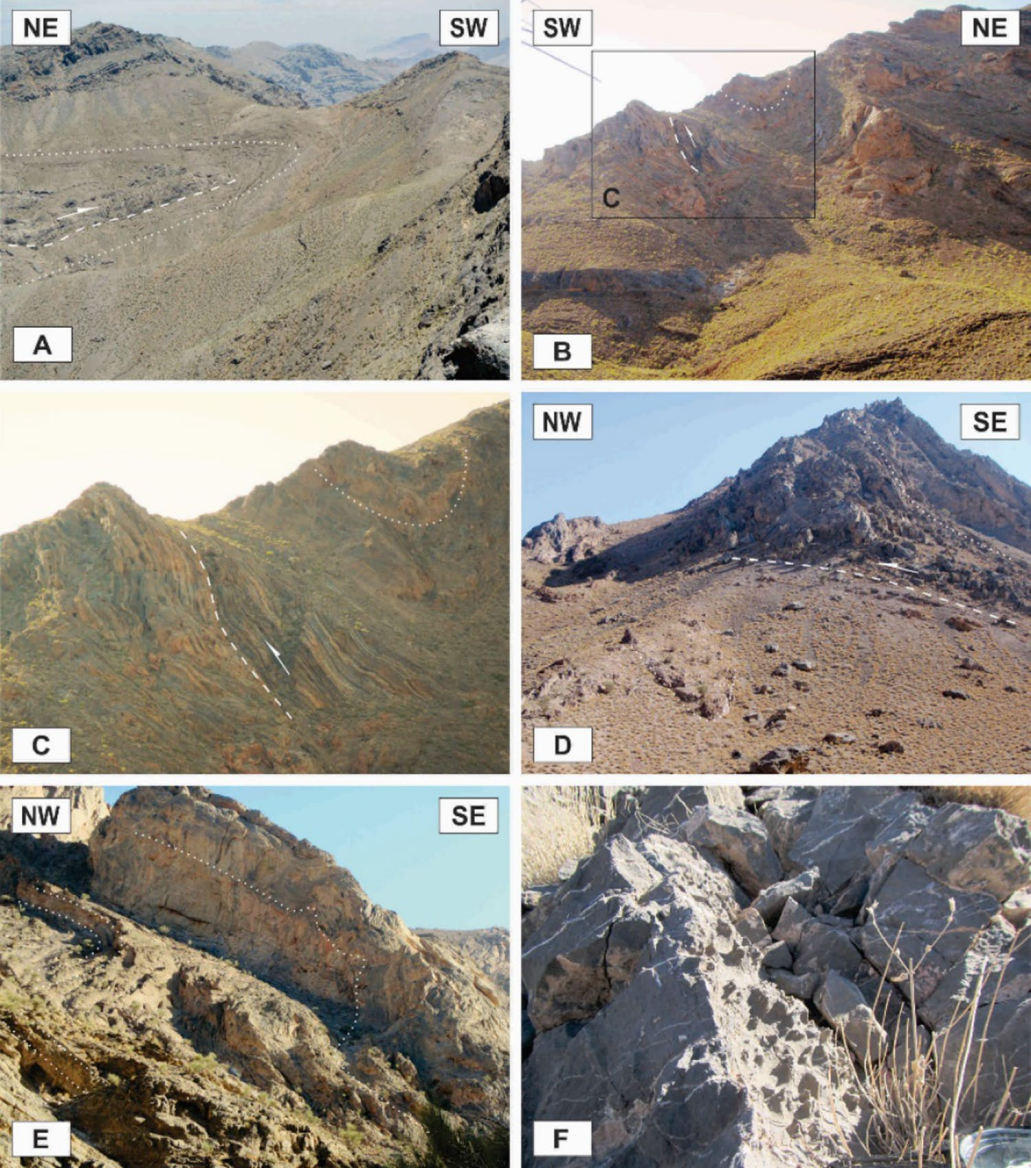
(**A–E**) Annotated field photographs of the several map-scale folds. (**F**) The tilted solution pits developed on the surfaces of the Permian limestone (P^l^) layers in the Faryadoun Mountain. The compass needle is 55 mm long and toward N. (Photos are taken by Mohammad Ali Ghanbarian).

The different kinds of map-scale faults are the most important structures in the region. It seems that the Dare-Nar valley has been formed due to an NNE–SSW tear fault. The change in the attitude of the layers on both sides of the Dare-Nar valley and the sharp scarp on the northwestern side of the valley confirm the existence of this tear fault, which is named here the Dare-Nar tear fault (Figs. 2, 3). The two most notable types of map-scale faults are thrusts (or reverse faults) and strike-slip faults, which are generally WNW–ESE and NW–SE striking, respectively. The strike-slip faults (Fig. 7A) have been more developed in the south of the study area (i.e., Faryadoun Mountain), while the thrust and reverse faults (Figs. 5, 7B–F) have been more developed in the north of the study area (i.e., Sorkhouy Mountain and Assouk Mountain). The general tectonic vergence of the region is to the SW (Sarkarinejad and Ghanbarian, 2014). Despite the existence of several considerable back thrusts (i.e., SW dipping; Fig. 7B–F), most of the thrusts in the area are NE dipping, which brought the different units from NE to SW (Fig. 5C–G). The bends in the faults’ surfaces resulted in flat-ramp-flat geometries, and these geometries, in turn, caused folding in the hanging wall blocks (fault-bend folds; Fig. 5C). There is also a map-scale into-anticline thrust in the Assouk anticline (Fig. 8A). The strike line of this steeply dipping reverse fault is NW–SE, which is parallel to the axial plane of the Assouk anticline and its dip is toward the NE. Even though the strikes of most of the strike-slip faults are N40W, there are some such faults with strikes ranging between N20–70° W in the Faryadoun Mountain (Fig. 3). The strike-slip faults are vertical or semi-vertical and dextral, while the dip of most of the thrusts and reverse faults is 10°–40°.

### Topographic step

A considerable topographic step is also recognizable between the Faryadoun Mountain and its northern area (Dareh-Nar valley and its adjacent areas, i.e., Assouk and Sorkhouy mountains). The average elevations in the north and south of this topographic step on the plotted profiles are 2010 and 2700 m, respectively (Fig. 6). This elevation contrast confirmed that there is a meaningful topographic step between the Faryadoun Mountain in the south and the Dareh-Nar valley in the north. The best explanation for the existence of this topographic step is the activity of a hidden basement SW-dipping thrust. Considering the overall tectonic vergence of the study area, which is to the SW, the NE verging basement fault is a back thrust. This inferred basement fault is named the Dareh-Bagh thrust.

### Map-scale folds

In the study area, there are several map-scale symmetric and asymmetric folds formed by the Devonian to Triassic rocks (Figs. 2, 3, 8A–E). They have occurred in the hanging wall and footwall blocks of the map-scale faults in which their axes or hinges are parallel or semi-parallel to these faults’ strike (NW–SE, Fig. 3). According to the diverse dips of their axial surfaces and the small plunge of their hinges, these macroscopic folds are horizontal inclined to upright and their profiles show that they are tight to open and even gentle folds. There are some tilted developed solution pits on the surfaces of the Permian limestone layers in the Faryadoun Mountain (Fig. 8F). The Assouk anticline (Fig. 3) is the most prominent fold in the study area, which is a gently plunging, steeply inclined, tight fold between the Faryadun mountain in the south and the Assouk mountain in the north. The Darreh-Bagh anticline and Darreh-Bagh syncline, which have been shown on the geological map of Kuh-e-Faryadon^34^, have not been recognized in this study.

### Outcrop-scale folds

There are lots of classes of mesoscopic folds at different scales with small wavelengths in the range of 0.02–10 m. The exposed outcrop-scale folds are seen more in the less erodible units such as Carboniferous and Permian limestones than in the Upper Devonian metaterrigenous unit and Lower Triassic marls and limestones. These mesoscopic symmetric and asymmetric folds have chevron, concentric, and box profiles and mostly consist of thin-bedded limestones and dolostones.

**Figure 9:**
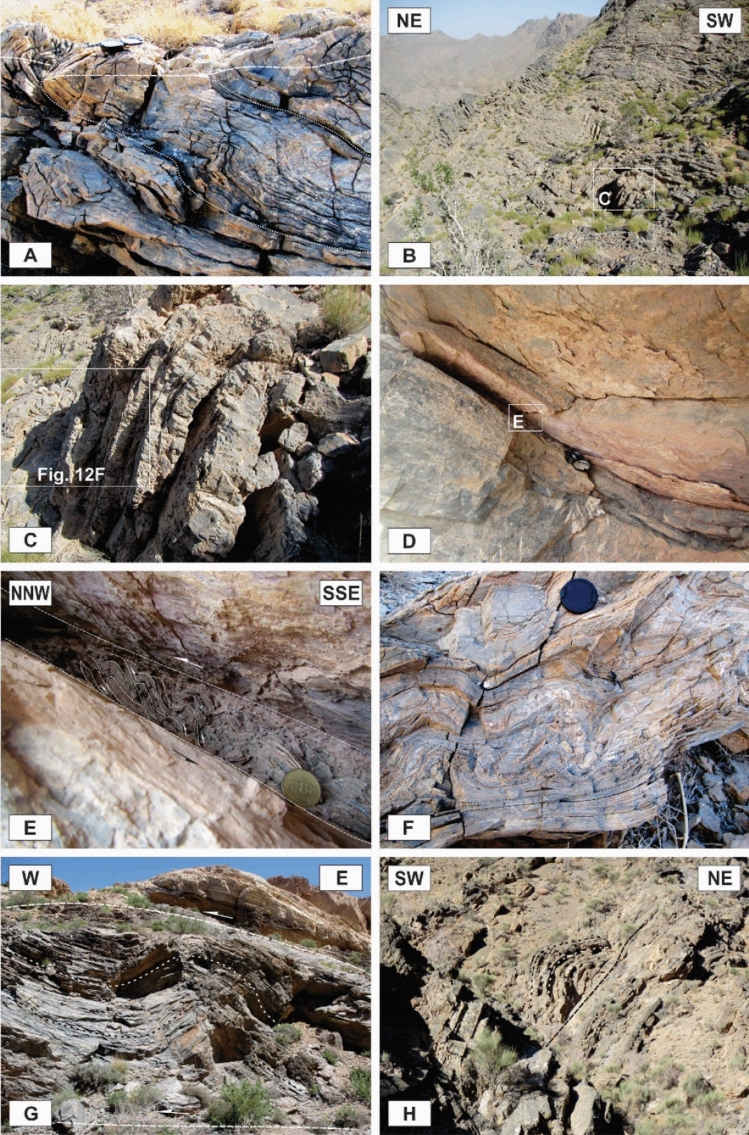
Annotated field photographs of the various kinds of the outcrop-scale folds in the different parts of the study area. (Photos are taken by Mohammad Ali Ghanbarian).

**Figure 10:**
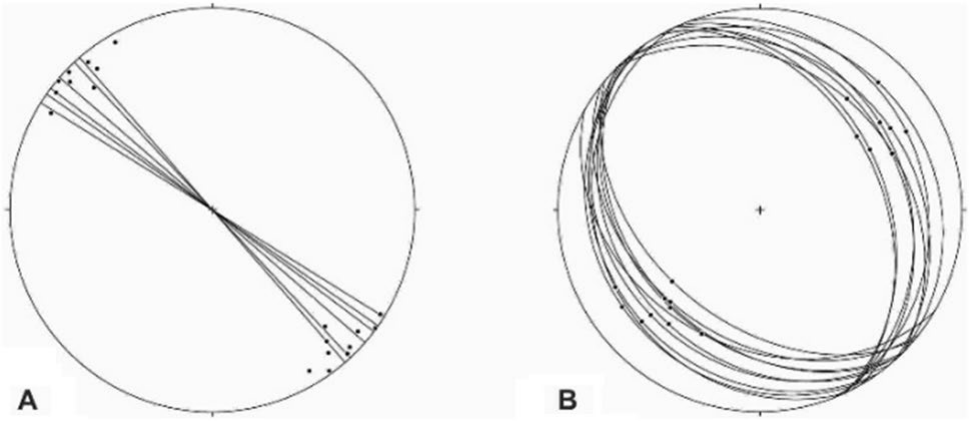
(**A**) Stereographic projection from the lower hemisphere showing the trend of map-scale folds (lines; No. = 7) and the attitudes of the axes of the outcrop-scale folds (points; No. = 19). (**B**) Stereographic projection from the lower hemisphere showing the attitudes of the outcrop-scale thrusts (No. = 18).

The axial planes are generally moderately to steeply dipping (50°–70°), although shallowly dipping (Fig. 9A) and vertical axial planes also occur. Despite the existence of different folds such as upright to recumbent and horizontal to vertical folds, the horizontal inclined folds are the more abundant (Fig. 9B,C). The interlimb angles vary greatly, but most of the outcrop-scale folds in the study area are too tight to open. They are located in the tilted layers, the limbs of the map-scale folds, and even in the brittle-ductile shear zones associated with small-scale faults in these zones (Fig. 9D,E). The trends of the folds’ axes, which are located on the tilted layers and the limbs of the map-scale folds, are mostly similar to the map-scale folds (the trends of their low plunge hinge lines are commonly toward NW or SE), which are semi-parallel to the strike of most of the thrust faults of the area (Fig. 10A); while the trends of the folds that are located in the brittle-ductile shear zones depend on the attitude and the sense of movement of the shear zones, the trends of their axes are generally normal to the displacement vectors of the shear zones. In addition, there are some outcrop-scale detachment (decollement) folds (Fig. 9F,G) which developed when displacements along bedding-parallel detachment faults (with no ramp) have been transferred into the folding of the hanging wall blocks. In the southern part of the Dare-Nar valley, there are some tilted (~ 50°) outcrop-scale detachment folds (Fig. 9H) due to thrusting and folding.

### Outcrop-scale faults

**Figure 11:**
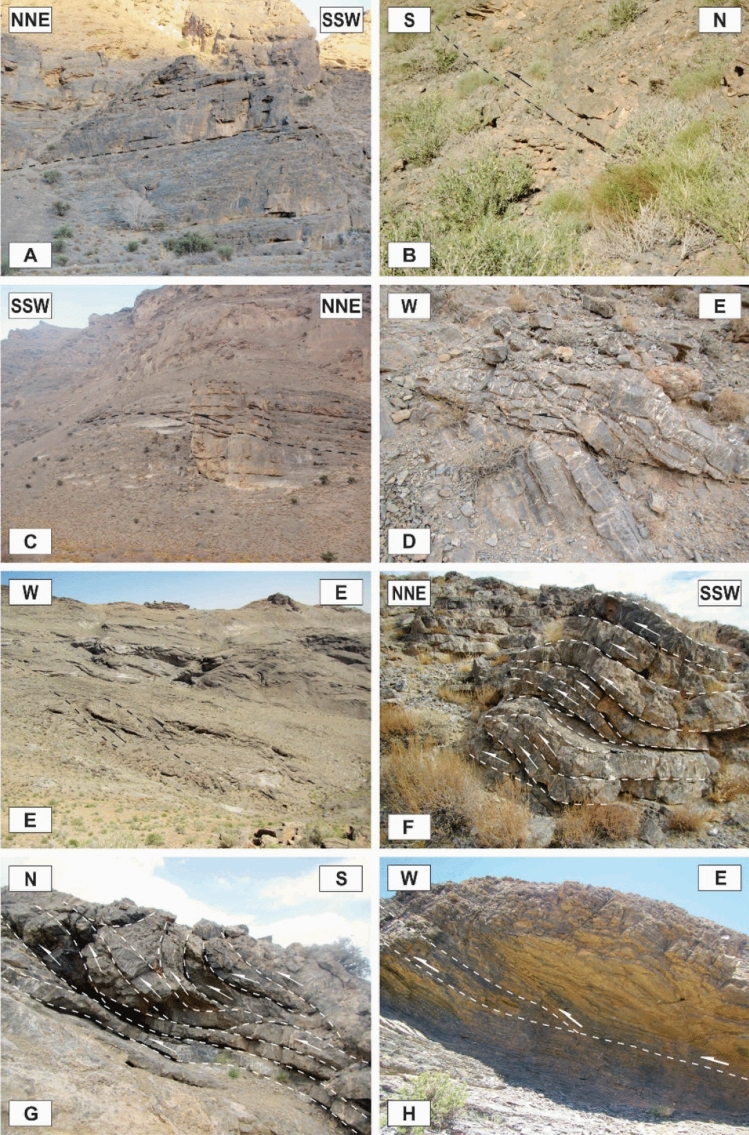
Annotated field photograph of the examples of the outcrop-scale thrust faults and duplex structures. (Photos are taken by Mohammad Ali Ghanbarian).

**Figure 12:**
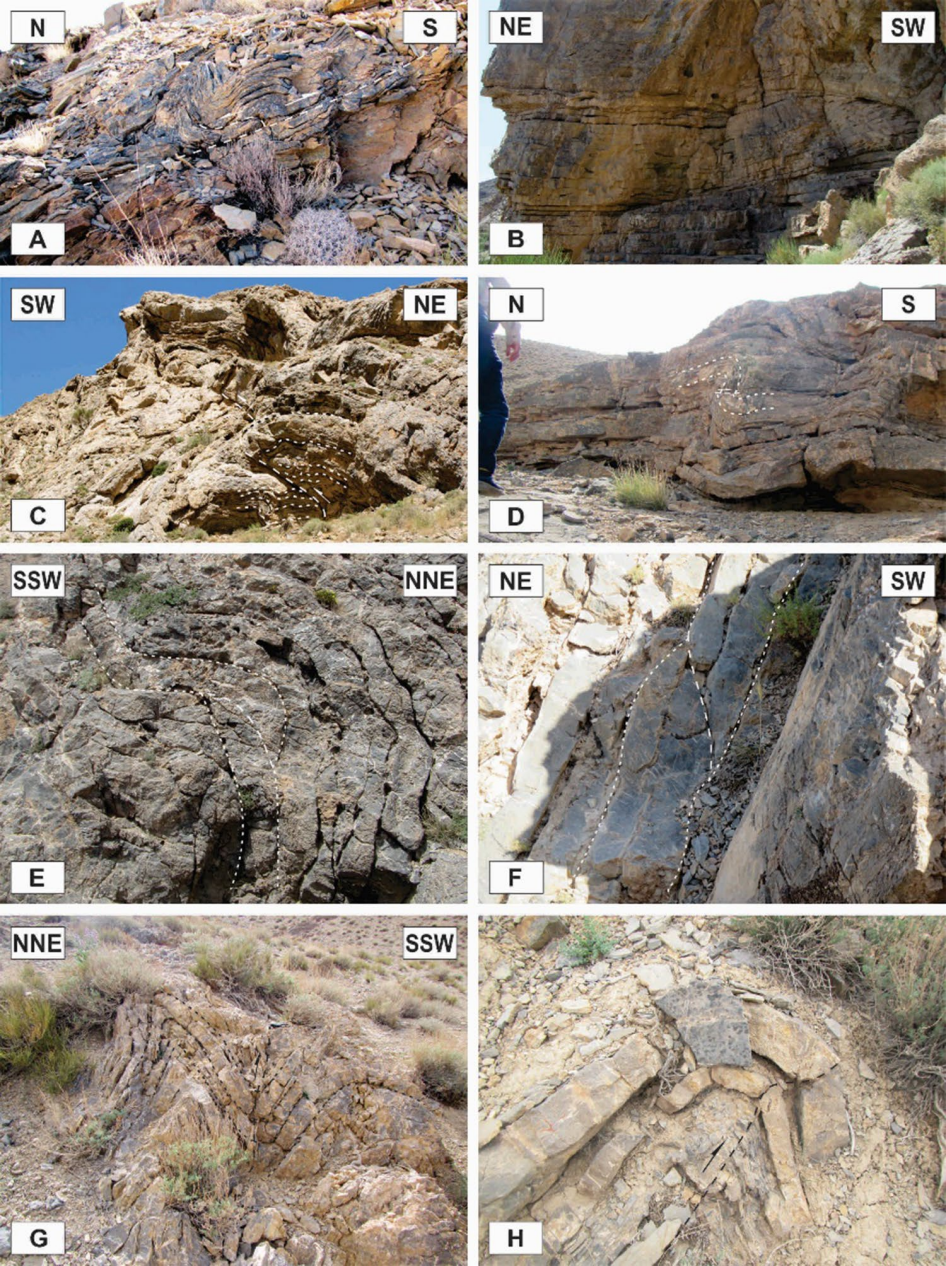
Annotated field photograph of some of the outcrop-scale fold-accommodation faults. (**A**) and (**B**) Back thrusts. (**C**) A Forelimb thrust. (**D**) A forelimb space-accommodation thrust. (**E**) A hinge wedge thrust. (**F**) A limb wedge thrust. (**G**) An out-of-syncline thrust. (**H**) An into-anticline thrust. (Photos are taken by Mohammad Ali Ghanbarian).

**Figure 13:**
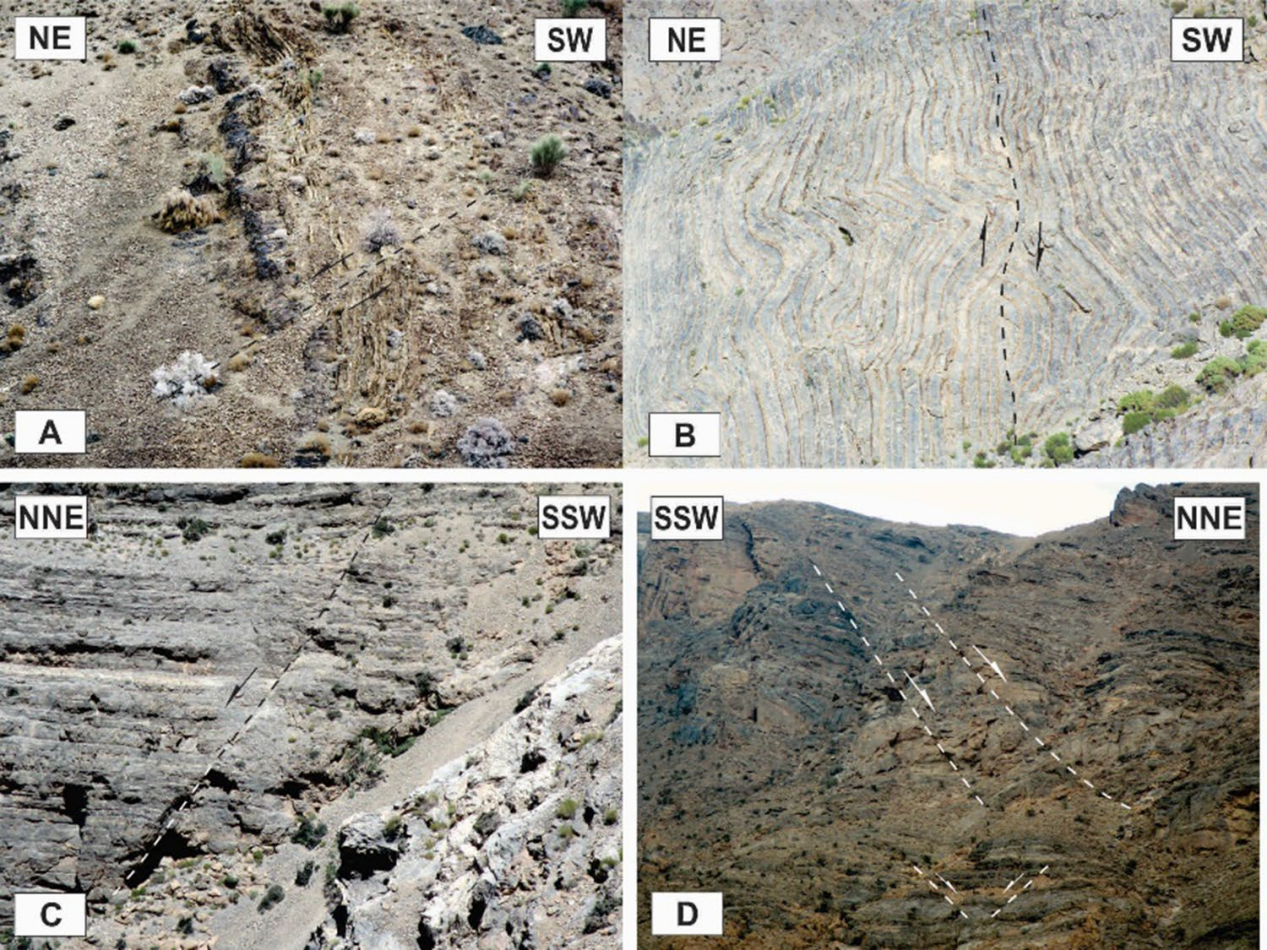
Annotated field photograph of the outcrop-scale strike-slip (**A**,**B**) and normal (**C**,**D**) faults. (Photos are taken by Mohammad Ali Ghanbarian).

**Figure 14:**
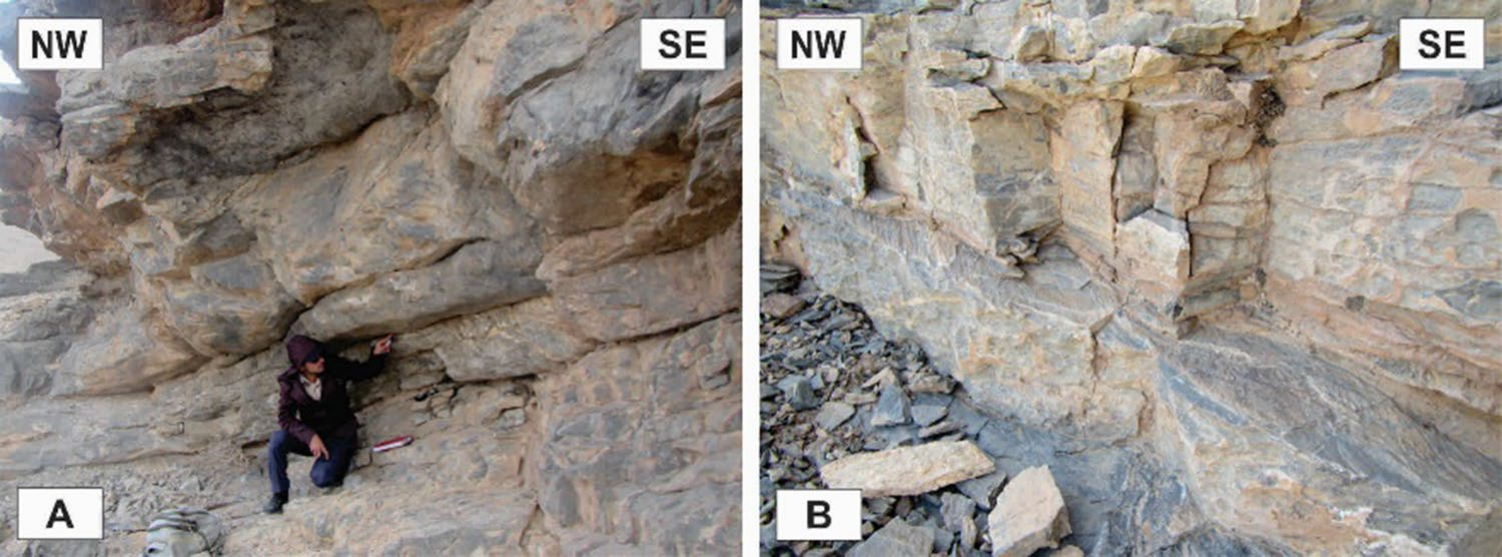
The slickensides on the contacts’ surfaces of the tilted layers in the limbs of the map-scale folds. (Photos are taken by Mohammad Ali Ghanbarian).

One of the most observed structures in the field investigations of the Faryadoun and Dareh-Nar areas is the outcrop-scale faults. There are different types of mesoscopic faults in the study area, such as various types of thrust (Figs. 10B, 11A–H, 12A–H), strike-slip (Fig. 13A,B), and normal faults (Fig. 13C,D). The outcrop-scale thrust faults are the most frequent ones, which occurred as the cause and/or result of the mesoscopic folds or without any obvious relationship to them. As a result of the activity of these numerous outcrop-scale thrust faults, some typical mesoscopic duplex structures formed (Fig. 11F,G). The hinge zones and limbs of the observed symmetric and asymmetric outcrop-scale folds have usually been dissected by mesoscopic thrust faults. The strikes of these thrust faults are mostly parallel to the hinge zones of the mesoscopic folds. These cm-displacement thrust faults cut only a few layers. As well as the different types of mesoscopic fault-related folds, especially fault bend folds (Fig. 11H), the various kinds of outcrop-scale fold-accommodation faults^7^, such as the back thrusts (Fig. 12A,B), the forelimb thrusts (Fig. 12C), the forelimb space-accommodation thrusts (Fig. 12D), the hinge wedge thrusts (Fig. 12E), the limb wedge thrusts (Fig. 12F), the out-of-syncline thrusts (Fig. 12G) and the into-anticline thrusts (Fig. 12H) are the common structures in the study area. There are a lot of slickensides on the contact surfaces of the tilted layers in the limbs of the map-scale folds (Fig. 14). The slickenlines are generally perpendicular to the folds’ axes.

Similar to the map-scale strike-slip faults, the outcrop-scale ones (Fig. 13A,B) are vertical to semi-vertical, often dextral, and more dominant in the southern parts of the study area (i.e., Faryadoun Mountain), with strikes that are generally similar to the other aforementioned faults (NW–SE). Outcrop-scale normal faults are the other obvious structures in the study area. The prevailing dips of these m-displacement faults are 55°–65°. Their attitudes vary considerably, but the E-striking ones are more frequent.

### Layer-parallel ductile shear zones

**Figure 15:**
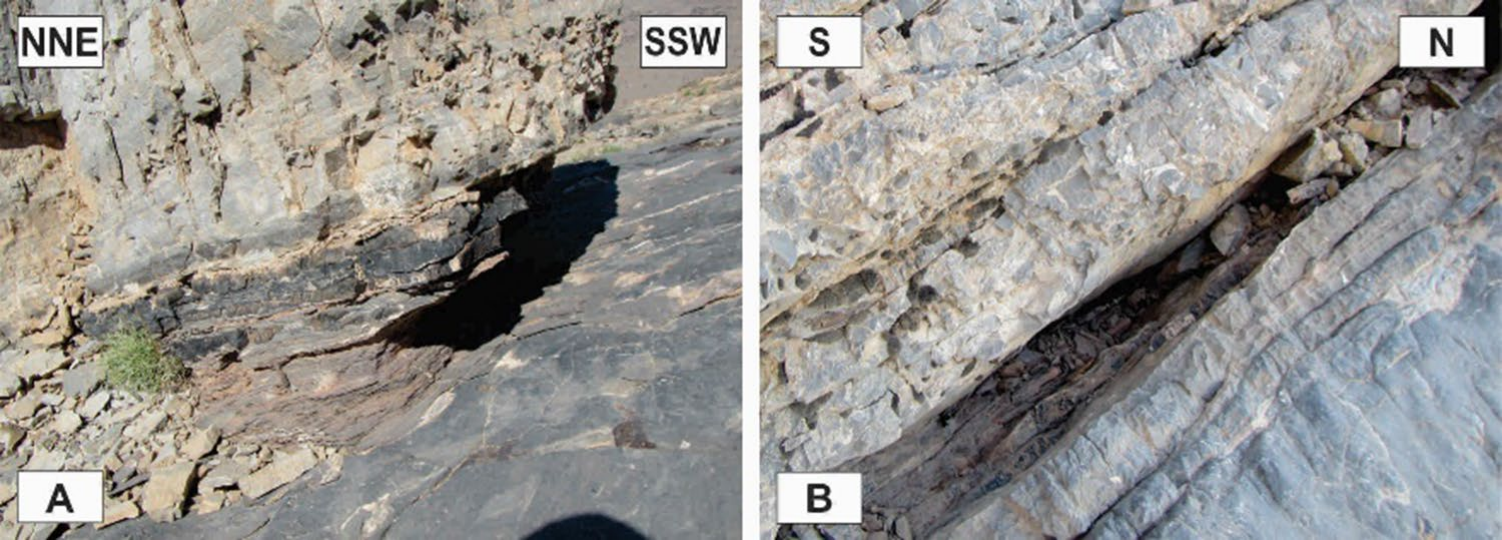
The Layer-parallel ductile shear zones. (Photos are taken by Mohammad Ali Ghanbarian).

In the Sorkhouy, Faryadun, and Assouk mountains, there are many layer-parallel narrow ductile shear zones. They are mostly NNW-SSE striking, ENE gently dipping in the Sorkhouy and Assouk mountains (Fig. 15A), and NW–SE striking, SE dipping in the Faryadun mountains (Fig. 15B). These shear zones have been formed in the less competent layers and have facilitated the shearing of the layers. They consist of layers of parallel calcite mylonitic foliation. Deformed chert nodules are plentiful in these shear zones (Fig. 15B). These less competent layers have been eroded more than the other layers (Fig. 15).

### Sinistral top-to-the NW deformation

**Figure 16:**
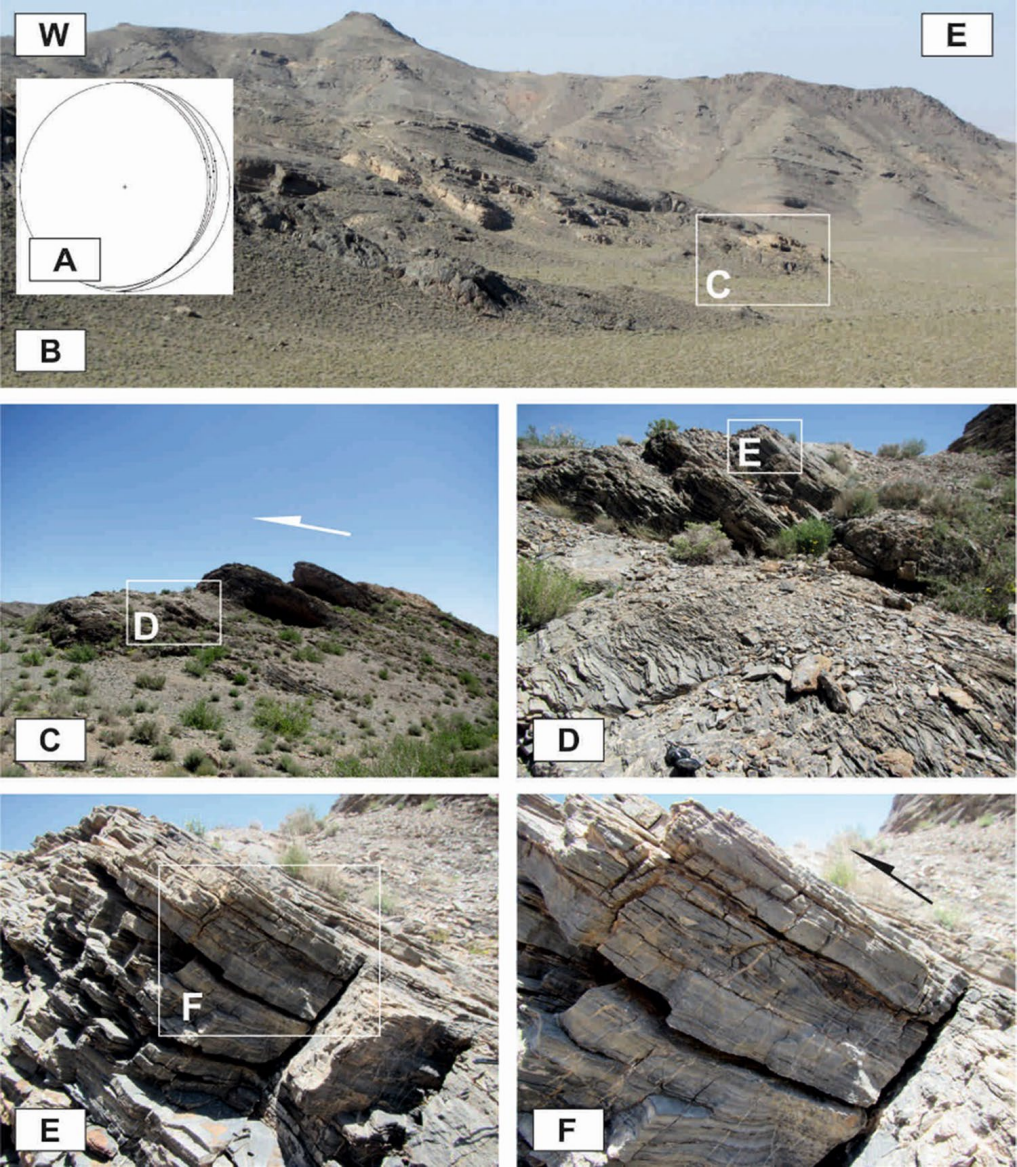
The top-to-the NW ductile deformation in the study area. (Photos are taken by Mohammad Ali Ghanbarian).

The field observations (Fig. 16) in terms of the fabric elements (Fig. 16A) and reliable shear sense indicators (Fig. 16B–F) reveal that there is obvious general sinistral top-to-the NW ductile deformation in the different parts of the study area^15,30,35–38^.

## Discussion

The SSZ is the inner part of the Zagros collisional zone which is deformed severely^39^. It has been developed as a consequence of the subduction of the Neotethyan oceanic plate under the Central Iranian microplate in the Jurassic to Cenozoic and the subsequent collision between this and the Arabian plate in the middle of the Cenozoic^20,39^. The SSZ has been divided into the southwestern metamorphic zone (i.e., SSMB) and the northeastern metasedimentary belt (i.e., ZHFTB)^14,30^. Almost all of the study area, which is the south-central part of the ZHFTB, is affected by low-grade metamorphism, except the units younger than the Early Eocene. According to the age dating results of the Sanandaj-Sirjan Zone^40^, the metamorphism of the area is considered to be older than the Eocene. There is at least one sinistral top-to-the NW shearing (Fig. 16)^30,35,36,38^ which is older than Neogene^40^. This sinistral top-to-the NW deformation is not restricted to this area^15,41^. The fabric of Heneshk (Kowlikosh) shear zones (Fig. 12 in^42^) and the Neyriz area (Fig. 3 in^43^), also, indicate the occurrence of sinistral deformation in the different parts of SSMB.

In the ZHFTB, the two architectural structures are faults and folds. In addition to the exposed macroscopic faults and folds, which have been presented on the maps (Figs. 2, 4), two hidden faults have been introduced in this study: the Dare-Nar tear fault along the linear Dare-Nar valley (Figs. 2, 3), and the Dare-Bagh back thrust, which resulted in a prominent topographic step in the north of the Faryadun mountain. The effects of hidden basement faults on the surface topography have not been presented before in this area but have been reported in many other areas, such as the Darang and Surmeh anticlines in the Zagros Foreland Folded Belt^44^. The cause of the existence of the Dare-Nar valley seems to be a NE-SW striking tear fault (i.e., the Dare-Nar tear fault), similar to the Talaee tear fault, which was introduced by Sarkarinejad and Ghanbarian^14^. The vertical offset, which is caused by the Dare-Nar tear fault, is much less than the offset of the Talaee tear fault. The relationships of the faults and folds in the outcrop-scale fault-bend folds and fold accommodation faults are more obvious than their association in the map-scale structures. The map-scale thrust faults on the limbs of the map-scale folds, however, suggest that these folds could develop as thrust-related folds^45,46^. Thus, the fault-related folds were observed at different scales and in the various types of fault-bend folds and asymmetric detachment folds. Several incompetent layers, such as the Upper Devonian metaterrigenous unit (D^sh^), the Lower Permian sandstone and shale (P^s^), and the Lower Triassic thinly-bedded marl and limestone (Tr^l^), acted as the detachment surfaces and facilitated occurrences of the thrust systems, detachment folds, and layer parallel shear zones. There is no evidence of a map-scale roof thrust, so the thrust systems are mostly imbricate fans. This is in contrast with the idea of Sarkarinejad and Ghanbarian^14^, which suggested that the structural architecture of the area is characterized by several duplex structures. Several successive map-scale foreland dipping horses, however, occurred in the center of the study area and north of the Faryadun mountain (Fig. 3). Therefore, the thrust faults play the most determining roles in the structural architecture of the study area, and many other structures, such as folds, have occurred as a result of their development. Sarkarinejad and Ghanbarian^14^ emphasized the determining role of the map-scale forethrusts, too. The map-scale back thrusts of the study area (Fig. 7C–F), which are very significant structures in the area (Fig. 3), can occur due to the flat-ramp geometry of a main underground fore thrust, the occurrence of pop-up structures, and the steepening of the underlying foreland-dipping duplexes (due to ongoing shortening of the area), which bend the flat thrusts to the SW-dipping thrust. These back thrusts can also be the continuation of the Dareh-Bagh basement back thrust, which caused the main topographic contrast in the vicinity of the main exposed back thrusts of the study area (i.e., the north of the Faryadun mountain; Fig. 3). Within the folds of the study area, there are different kinds of faults that have developed due to folding processes. Forelimb thrusts, forelimb space-accommodation thrusts, back thrusts, out-of-syncline and into-anticline thrusts, wedge thrusts including hinge wedges and limb wedges thrusts are the diverse modes of the fold accommodation faults of the study area. Nevertheless, the geometric and kinematic relationship between thrust faults and folds are scale-invariant, as discussed by Sarkarinejad and Ghanbarian^14^. The existence of many slickensides between tilted layers in the macroscopic folds’ limbs suggests that there are layer parallel slips that developed during flexural slip folding.

The Upper Oligocene–Lower Miocene reefal limestones are involved in younger than the earliest Miocene thrusting. The tilted solution pits on the surfaces of the Permian limestone layers in the Faryadoun Mountain (Fig. 8F) indicate that the fault-related folding in the area is very young, too. Some dextral strike-slip faults have cut the northern parts of Faryadoun Mountain (Fig. 3), a phenomenon that is well-documented by Nadimi and Konon^47^ in the Esfahan region. There is no apparent kinematic relationship between the strike-slip faults and folds.

Therefore, there must be at least two major events, (1) a ductile one older than the Eocene, which is accompanied by metamorphism and sinistral top-to-the NW deformation, and (2) a semi-brittle deformation younger than the earliest Miocene. The latter is represented by folding, thrusting, and strike-slip faulting. The folding and faulting in this event are interrelated, as shown by the existence of various fault-related folds and fold-related faults in the area. This kinematic association suggests that this second deformation event is not completely brittle.

## Conclusion

The structural analysis of this part of the ZHFTB was based on aerial photographs stereoscopy, satellite (Google Earth) images interpretations, consideration of the major topographic changes, and field investigations. The results of this research revealed that there must be at least two main deformation phases, (1) a ductile event, which is older than Eocene and accompanied by metamorphism and sinistral top-to-the NW deformation, and (2) a semi-brittle deformation phase that is younger than the earliest Miocene and is represented by thrusting, folding, and strike-slip faulting. The abundant fault-related folds and fold-accommodation faults of the study area reveal the close connection between folding and faulting in this region. A basement hidden back thrust and a major hidden tear fault has been introduced in this study based on the surface topographic changes. The basement hidden back thrust may be the cause of the map-scale back thrusts and other structures in the north of Faryadun Mountain.

## Data Availability

All data generated or analysed during this study are included in this published article.
